# A Comprehensive Study Employing Computational Analysis and Mendelian Randomization Has Revealed the Impact of Key Genes on Liver Cancer

**DOI:** 10.3390/biomedicines13061313

**Published:** 2025-05-27

**Authors:** Size Li, Wenying Qi, Junzheng Wu, Chunhua Luo, Shihao Zheng, Xu Cao, Wei Wang, Qiyao Liu, Hongbo Du, Xiaoke Li, Xiaobin Zao, Yongan Ye

**Affiliations:** 1 Dongzhimen Hospital, Beijing University of Chinese Medicine, Beijing 100700, China; 2 Xiamen Hospital of Traditional Chinese Medicine, Xiamen 361006, China; 3 Key Laboratory of Chinese Internal Medicine of Ministry of Education and Beijing, Dongzhimen Hospital, Beijing University of Chinese Medicine, Beijing 100700, China; 4 Liver Diseases Academy of Traditional Chinese Medicine, Beijing University of Chinese Medicine, Beijing 100700, China

**Keywords:** hepatocellular carcinoma, EHD4, PPARGC1A, Mendelian randomization, immune cell infiltration, tumor-associated macrophages

## Abstract

**Background and Aims**: In this research, we sought to enhance our comprehension of liver cancer’s genetic architecture by employing Mendelian randomization (MR) techniques to establish causative relationships between particular genetic variations and liver cancer susceptibility. **Methods**: We integrated data from the public databases with MR analysis to identify differentially expressed genes (DEGs) associated with Hepatocellular Carcinoma (HCC). We conducted functional enrichment analyses to determine the biological processes and signaling cascades associated with the identified DEGs. We also used the CIBERSORT deconvolution method to evaluate immune cell composition in HCC tissues, followed by correlation studies examining relationships between our key genes of interest and various immune cell populations. Additionally, we validated our findings using a rat model of HCC and clinical HCC samples. **Results**: We obtained two key genes, *EHD4* and *PPARGC1A*, which co-regulated M0 macrophages, suggesting their role in macrophage polarization and tumor progression. In addition, *PPARGC1A* is associated with resting and activated mast cells, suggesting its involvement in regulating the tumor microenvironment. Detection of rat and clinical samples further confirmed the upregulation of these genes in HCC, supporting their potential as therapeutic targets. **Conclusions**: Our findings emphasize the significant involvement of *EHD4* and *PPARGC1A* in HCC, specifically regarding their influence on tumor-associated macrophage polarization and broader immune microenvironment modulation. These findings offer new insights into the molecular mechanisms driving HCC and suggest that targeting these genes may provide novel strategies for personalized treatment.

## 1. Introduction

Globally, primary liver malignancies now rank as the third leading cause of cancer-related mortality, claiming roughly 0.83 million lives each year [1]. Hepatocellular carcinoma (HCC) dominates the histological spectrum, representing about 85–90% of primary hepatic malignancies [2]. Even with better screening and therapy, the five-year survival seldom exceeds 20%, dropping to roughly 10% where healthcare access is scarce [3]. The risk profile is shifting: besides viral hepatitis, metabolic dysfunction-associated steatotic liver disease (MASLD) and alcohol-induced injury are now prominent drivers of HCC [4]. Large-scale genomics highlight recurrent alterations in the *TERT* promoter, *TP53*, *CTNNB1*(β-catenin), and *AXIN1*, which, together with environmental insults, propel hepatocarcinogenesis [5].

Curative resection or ablation benefits early HCC, yet high rates of relapse and metastasis persist owing to intratumoral heterogeneity [6]. In advanced disease, the multikinase inhibitor sorafenib prolongs survival but remains financially inaccessible to many patients [7]. Prognostic biomarkers are pivotal for tailoring therapy and optimizing clinical decision-making.

Recent epidemiological and genomic studies have consistently demonstrated that liver cancer exhibits significant genetic susceptibility. A large cross-sectional study in Sweden found that individuals with a first-degree relative who had developed HCC had a standardized incidence ratio (SIR) of 2.60 for liver cancer [8]. In the Nordic Twin Study, a familial clustering of liver cancer was observed, with an estimated familial risk of liver cancer in monozygotic twins of 2.1%, which is higher than in the general population [9]. In 2024, a large-scale genome-wide association study (GWAS) conducted across multiple centers in North America identified key genetic loci significantly associated with non-viral HCC, including *PNPLA3*rs738409 C>G (I148M), *TM6SF2*rs58542926 C>T (E167K), and *MAU2* rs58489806 C>T [10]. In the same year, a transcriptomics-associated study based on a joint Chinese–Japanese cohort identified 22 candidate susceptibility genes at 16 new loci, indicating the presence of a unique genetic landscape in East Asian populations [11].

Currently, most studies are limited to statistical associations and lack causal inference and functional validation. This study combines MR analysis, bioinformatics analysis, animal experiments, and clinical sample detection to explore the molecular mechanisms of HCC, identify potential intervention targets, and provide a reference for the precision treatment of HCC.

## 2. Materials and Methods

### 2.1. Raw Data Acquisition

We acquired transcriptomic profiles and corresponding clinical data for GSE14520 and GSE84402 cohorts through the NCBI Gene Expression Omnibus repository. We collected expression quantitative trait loci (eQTL) information for 19,943 genes to serve as exposure parameters from the MRC IEU OpenGWAS database. We extracted liver cancer data ieu-b-4953 from the ieu database and used it as an outcome indicator for our analyses. We employed UCLCAN (https://ualcan.path.uab.edu) (accessed on 10 December 2024)to obtain comprehensive data on *EHD4* mRNA expression in tumor samples, corresponding paracancerous tissues, and normal controls. The protein expression data of EHD4 were obtained from the HPA (http://www.proteinatlas.org) (accessed on 10 December 2024) and CPTAC (https://proteomics.cancer.gov) (accessed on 10 December 2024) databases.

### 2.2. Differential Analysis

The GSE14520 and GSE84402 cohorts were combined, yielding 234 non-tumor and 239 HCC samples. Differential expression was assessed with the limma package (version 3.58.1) in R 4.4.2, adopting an adjusted-P (FDR) < 0.05 and |log₂ fold-change| > 0.585 as cut-offs for significance. Results were depicted as a volcano plot (ggplot2) and a clustered heatmap to highlight the most prominent expression shifts.

### 2.3. Gene Set Enrichment Analysis

We implemented Gene Set Enrichment Analysis to identify functional categories and biological pathways associated with our co-expressed gene clusters, visualizing enrichment patterns at either end of the ranked gene list to indicate upregulated or downregulated processes. This analytical approach allowed us to investigate the activation status of various biological mechanisms within our experimental gene expression datasets. In GSEA analysis, a *p*-value less than 0.05 is considered statistically significant.

### 2.4. Inference of Infiltrating Immune Cells

For immune cell composition analysis, we employed the CIBERSORT computational framework, a validated deconvolution method that calculates immune cell proportions from bulk transcriptome profiles. After performing appropriate normalization of the HCC expression data, we determined the relative frequencies of 22 immune cell subpopulations across all samples in our study cohort. Following the determination of the immune cell profiles, we conducted correlation analyses to explore the relationships between these immune cell populations and the key genes previously identified in our study.

### 2.5. Instrumental Variable Selection

To identify potential causal relationships, we applied stringent criteria for selecting appropriate single-nucleotide polymorphisms (SNPs) as instrumental variables. Initially, we employed a genome-wide significance threshold (*p* < 5 × 10^−8^) to screen SNPs associated with metabolite biomarkers. To eliminate the influence of linkage disequilibrium (LD), we implemented LD clumping, excluding variants with an R² value greater than 0.001 or located within a ±10,000 kb window based on the European ancestry reference panel from the 1000 Genomes Project. Additionally, we calculated mean F-statistics to evaluate the robustness of our instrumental variables, adhering strictly to established methodological standards.

### 2.6. Instrumental Variable Analysis

For causal inference, we employed Inverse Variance Weighting (IVW), Weighted Median (WM), and MR-Egger regression methods. *p*-values were Bonferroni-adjusted for multiple comparisons, with associations deemed potentially causal at *p* < 0.05 and highly significant at *p* < 6.8 × 10^−5^. MR-Egger regression and MR-PRESSO were used to detect pleiotropy, while Cochran’s Q test evaluated heterogeneity. We implemented leave-one-out analysis to assess individual SNP influence on results. Effect sizes were reported as odds ratios (ORs) with 95% confidence intervals following standard epidemiological practices [12]. All analyses were conducted using R version 4.4.2, RStudio, and the “TwoSampleMR version 0.6.15 ” package.

### 2.7. Animals

Healthy male Wistar rats, specific pathogen-free (SPF) grade and weighing 170–200 g, were provided by Beijing Vital River Laboratory Technology Co., Ltd. (Beijing, China). (License No: SCXK (Jing) 2016-0006). This study was approved by the Ethics Committee of Experimental Animals of Dongzhimen Hospital, Beijing University of Chinese Medicine (Ethics Approval No. 21-10-01). The rats were conventionally housed in the barrier environment animal room of the Key Laboratory of Traditional Chinese Medicine Internal Science, First Clinical Medical College, Beijing University of Chinese Medicine (Batch No.: SYXK (Jing) 2015-0001), under controlled conditions of constant temperature (24 ± 1 °C), constant humidity (60 ± 10%), and a 12 h light–dark cycle, with free access to water and food. After a one-week acclimatization period, the rats were randomly assigned to groups, and the experimental procedures were initiated.

### 2.8. Design of Rat HCC Experiment

We selected the diethylnitrosamine (DEN)-induced HCC model because it replicates the progression from chronic inflammation to fibrosis, cirrhosis, and eventual cancer formation, similar to the clinical course seen in most HCC patients [13,14]. The HCC rat model was established by administering DEN (50 mg/kg/week, Psaitong, N60001, Beijing, China) intraperitoneally for sixteen consecutive weeks. The experimental animals were taken to observe the liver condition after the 16th week. Rats were fasted for 8 h before anesthesia, weighed, and anesthetized by intraperitoneal injection of 0.3% sodium pentobarbital solution at a dose of 0.2 mL/100 g. Abdominal aortic blood was collected, and the blood from each rat was centrifuged at 3000 rpm for 10 min. The serum was aspirated into cryovials, temporarily placed in a 4 °C refrigerator, and then transferred to a −80 °C freezer for preservation. After blood collection, the abdominal aorta was cut to drain the blood, and the liver was quickly removed. The tissue was separated in pre-cooled phosphate-buffered saline (PBS), wiped clean with absorbent paper, and photographed. The largest lobe of the liver was excised, and a tissue block (approximately 1 cm × 1 cm × 0.3 cm was cut from the liver approximately 1 cm away from the edge to retain the liver tissue and pathological specimens. The liver tissue for testing was placed in a cryovial, preserved in liquid nitrogen, and then transferred to a −80 °C freezer. Pathological tissues were placed in embedding boxes and fixed in a 4% paraformaldehyde solution for more than 48 h before processing. Finally, the rats were checked for their condition and euthanized by cervical dislocation.

### 2.9. Determination of Serological Indicators

The serological indicators alanine transaminase (ALT, OSR6107) and aspartate aminotransferase (AST, OSR6209) were detected using a Beckman Coulter AU Biochemical Analysis System (Beckman Coulter, Brea, CA, USA), and the sample concentration was automatically calculated.

### 2.10. Quantitative Real-Time PCR (qRT-PCR)

Following the manufacturer’s protocol, total hepatic RNA was isolated with the RaPure kit (Magen, R4011, Shanghai, China). One microgram of RNA was reverse-transcribed using the ABScript III mix (Abclonal, RK2029, Beijing, China) kit. qRT-PCR was carried out on a QuantStudio 6 Pro Real-Time PCR System (Thermo Fisher Scientific, Waltham, MA, USA) with SYBR Fast master mix (Abclonal, RK21203). The primer sequences used for qRT-PCR are listed in Table 1. Cycling parameters: 95 °C × 30 s; 40 cycles of 95 °C × 5 s and 60 °C × 30 s. Specificity was verified by melt-curve analysis. Relative expression was calculated by 2^−ΔΔCt^ using *GAPDH* as reference.

### 2.11. Liver Transcriptome Sequencing

Total RNA was isolated from Wistar rat liver using TRIzol^®^ reagent, with quality evaluated by 5300 Bioanalyzer (Agilent, Santa Clara, CA, USA) and quantified via ND-2000 (NanoDrop, Wilmington, DE, USA). Shanghai Majorbio (Shanghai, China) conducted RNA processing and sequencing following Illumina protocols. Four rat liver samples per group underwent differential expression analysis. Transcript levels were calculated using the transcripts per million (TPM) method with RSEM software1.3.3 for quantification. DEGs were identified through both DESeq2 and DEGseq platforms, applying thresholds of |log2FC| ≥ 1 and either FDR ≤ 0.05 (DESeq2) or FDR ≤ 0.001 (DEGseq).

### 2.12. Total Protein Extraction

Frozen liver (≈50 mg) was pulverized under liquid nitrogen and lysed in 8 M urea/1% SDS/protease inhibitor cocktail (1 mL). Homogenates were sonicated on ice (three 5 s bursts, 20 kHz) and cleared by centrifugation (13,000× *g*, 15 min, 4 °C). Protein concentration was determined with a bicinchoninic acid assay (ThermoFisher, 23225, Waltham, MA, USA).

### 2.13. Protein Expression Detection

Protein expression was quantified using the Simple Western capillary immunoassay platform (ProteinSimple, BioTechne, Minneapolis, MN, USA) with the following antibodies: *GAPDH* (CellorLab, LF205S, Beijing, China) at 1:50 dilution, *PPARGC1A* (Proteintech, 66369-1-Ig, Wuhan, China) at 1:200 dilution, and *EHD4* (Proteintech, 11382-2-AP, Wuhan, China) at 1:100 dilution. Other reagents were sourced from the ProteinSimple accessory kit (12–230 kDa Separation Module, 8 × 25 capillary cartridges, SM-W004). All analyses were performed using Compass software (v6.1.0). The grayscale values were analyzed using the ImageJ software (version 1.53t).

### 2.14. Clinical Samples

Three hepatocellular carcinoma (HCC) paraffin section samples were collected from the Xiamen Traditional Chinese Medicine Hospital. All samples were histopathologically confirmed with HCC criteria. The study protocol was approved by the Ethics Committee of Xiamen Traditional Chinese Medicine Hospital.

### 2.15. Histology Staining Analysis

The tissues were fixed overnight in 4% formalin (Servicebio, G1101, Beijing, China) and embedded in paraffin. Tissue sections were stained with hematoxylin and eosin (H&E) staining using a kit from Servicebio (G1003, Beijing, China) to observe the liver structure. Tissue sections were stained with Masson staining using a kit from Servicebio (G1006, Beijing, China) to observe the collagen production in the liver. Tissue sections were stained with periodic acid–Schiff (PAS) staining using a kit from Servicebio (G1008, Beijing, China) to observe the accumulation of glycogen in the liver. The quantification of the positively stained area was calculated by ImageJ software (version 1.53t).

### 2.16. Immunohistochemistry (IHC) Analysis

For IHC, the tissue sections were deparaffinized and rehydrated before antigen retrieval, removal of endogenous peroxidase, and blocking with normal goat serum. To detect proliferating cells, the Ki67 rabbit polyclonal antibody (GB111499, Servicebio, Beijing, China) was added dropwise with incubated in a wet chamber overnight at 4 °C. To detect *EHD4*, the paraffin-embedded sections were incubated overnight at 4 °C with the primary anti-*EHD4* (rabbit pAb, Proteintech, 13151-1-AP, Chicago, IL, USA). The next day, the reaction solution and HRP-labeled anti-rabbit secondary antibody (GB23303, Servicebio, Beijing, China) were added dropwise and incubated at 37 °C for 30 min. Diaminobenzidine (G1212, Servicebio, Beijing, China) was applied to provide a chromogen, referring in a reddish-brown color. Positive expression was defined as brown-yellow granules in the cytoplasm.

### 2.17. Immunofluorescent (IF) Staining

For IF, the tissue sections were deparaffinized and rehydrated before antigen retrieval, removal of endogenous peroxidase, and blocking with normal goat serum. To detect ARG1, the paraffin-embedded sections were incubated overnight at 4 °C with the primary anti-ARG1 (rabbit pAb, Servicebio, GB11285, Beijing, China). The next day, the reaction solution and Alexa Fluor 488-labeled anti-rabbit secondary antibody (GB25303, Servicebio, Beijing, China) were added dropwise and incubated at 37 °C for 30 min. DAPI dye solution was added (G1012, Servicebio, Beijing, China) and incubated at room temperature for 10 min. The sections were placed under a scanner for image collection. The nucleus stained by DAPI is blue under ultraviolet excitation, and the positive expression is green light labeled by the corresponding fluorescein.

## 3. Results

### 3.1. Differentially Expressed Gene (DEG) Analysis

**Figure 1:**
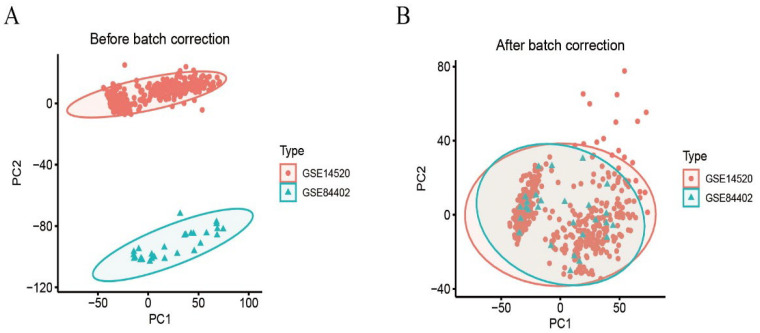
Dataset calibration. (**A**): PCA analysis before batch correction; (**B**): PCA analysis after batch correction.

**Figure 2:**
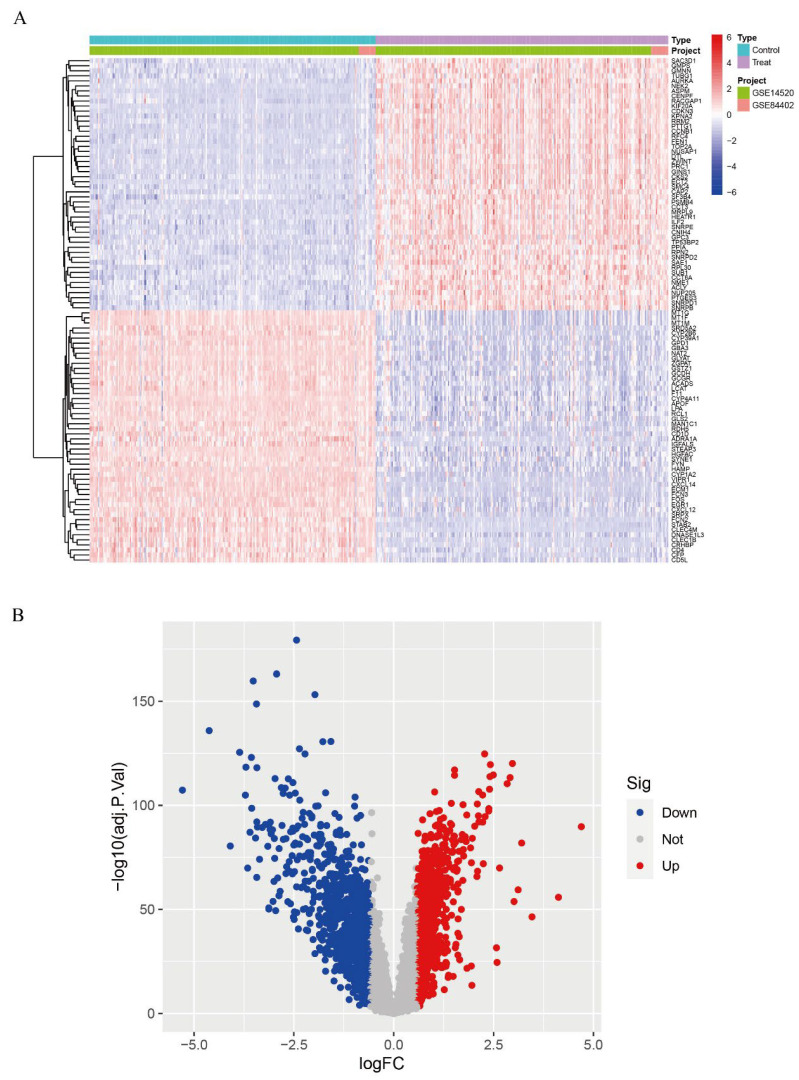
Differential gene expression analysis. (**A**): Expression heatmap of DEGs; (**B**): Volcano plot of DEGs.

We calibrated and integrated the expression values of each gene across their respective datasets using R version 4.4.2 and eliminated batch effects through principal component analysis (PCA). Initial analysis revealed batch effects in the two HCC gene datasets (Figure 1A); however, after correction and PCA, all the samples achieved acceptable homogeneity (Figure 1B). Comparative profiling of tumor versus non-tumor liver tissue in GSE14520 and GSE84402 datasets revealed 1831 differentially DEGs—928 up-regulated and 903 down-regulated. Figure 2A displays the 50 most strongly induced and repressed transcripts in a heatmap, whereas Figure 2B depicts their overall distribution in a volcano plot.

### 3.2. The Hub Genes Analysis

**Figure 3:**
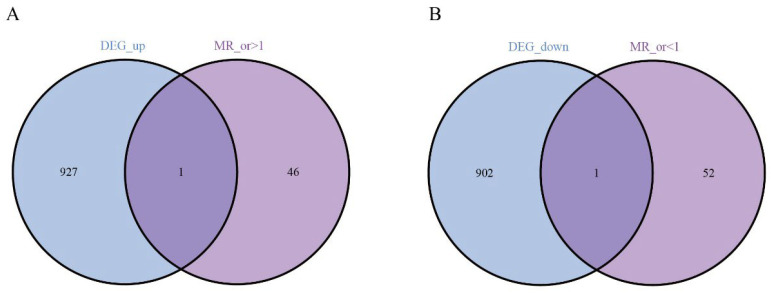
Analysis of key genes. (**A**): The Venn analysis of up-regulated and MR_ gene; (**B**): 1 down-regulated co-expressed gene.

Next, we performed Mendelian randomization (MR) analysis and obtained the genes with or >1 and or <1. Intersecting these DEGs with the genes highlighted by our screen pinpointed two central candidates—*EHD4* and *PPARGC1A* (Figure 3).

### 3.3. Causal Relationship Between the Hub Genes and HCC

**Figure 4:**
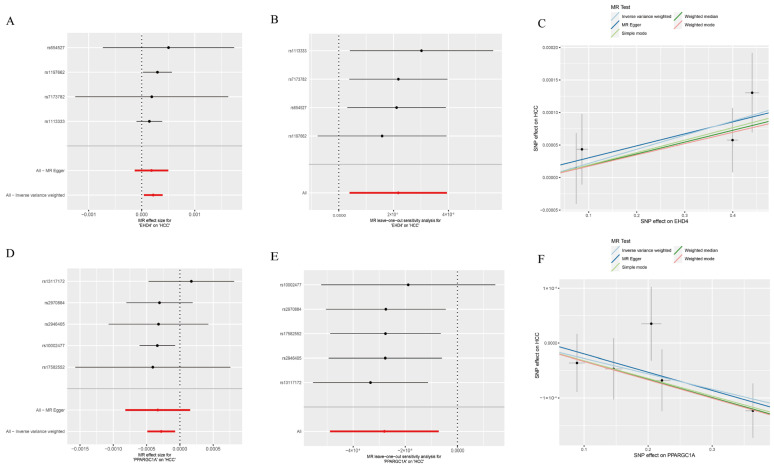
Mendelian randomization analysis of the hub genes. (**A**): Forest plot of *EHD4* against HCC risk; (**B**): Leave-one-out test for *EHD4* on HCC; (**C**): Individual estimates of the causal effect of *EHD4* on HCC; (**D**): Forest plot of *PPARGC1A* on HCC risk; (**E**): *PPARGC1A* leave-one-out test for HCC; (**F**): Individual estimates of the *PPARGC1A* causal effect on HCC. In panels (**A**,**B**,**D**,**E**): Red lines represent the overall MR effect estimates, while black lines represent individual SNP effect estimates with their confidence intervals.

**Figure 5:**
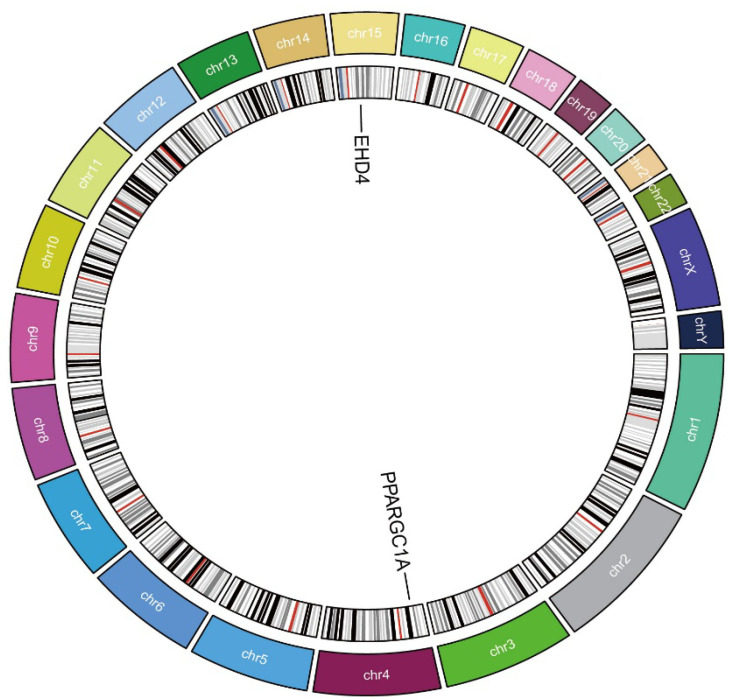
Circos plot of the hub genes. The circular diagram shows chromosomal locations, genomic positions, and functional connections between identified genes. Different colors represent distinct chromosomes.

We next employed two-sample MR to explore whether the two HCC-related hub genes exert a causal influence on tumor risk. Using the inverse-variance-weighted (IVW) estimator, *EHD4* displayed a significant positive association with HCC (Figure 4A–C), whereas *PPARGC1A* was inversely associated with disease susceptibility (Figure 4D–F). Neither the Cochran Q test for heterogeneity nor MR-Egger’s intercept for horizontal pleiotropy reached statistical significance (*p* > 0.05), indicating that our findings were unlikely to be confounded by variant heterogeneity or directional pleiotropy. Leave-one-out analyses further confirmed that removal of any single instrumental variable (IV) had only a minimal impact on the pooled causal estimate, underscoring the robustness of the results (Supplementary Materials). Finally, to illustrate the genomic context of these loci, we plotted the chromosomal positions of all co-expressed genes (Figure 5). The Circos plot demonstrates that *EHD4* and *PPARGC1A* are located on different chromosomes but show significant functional interactions through co-expression networks. This genomic mapping reveals potential regulatory hotspots and highlights chromosomal regions that may play critical roles in HCC pathogenesis. The interconnecting lines between different genomic locations suggest potential trans-regulatory mechanisms that could coordinate the expression of these genes during hepatocarcinogenesis.

### 3.4. Gene Set Enrichment Analysis (GSEA) of the Hub Genes

**Figure 6:**
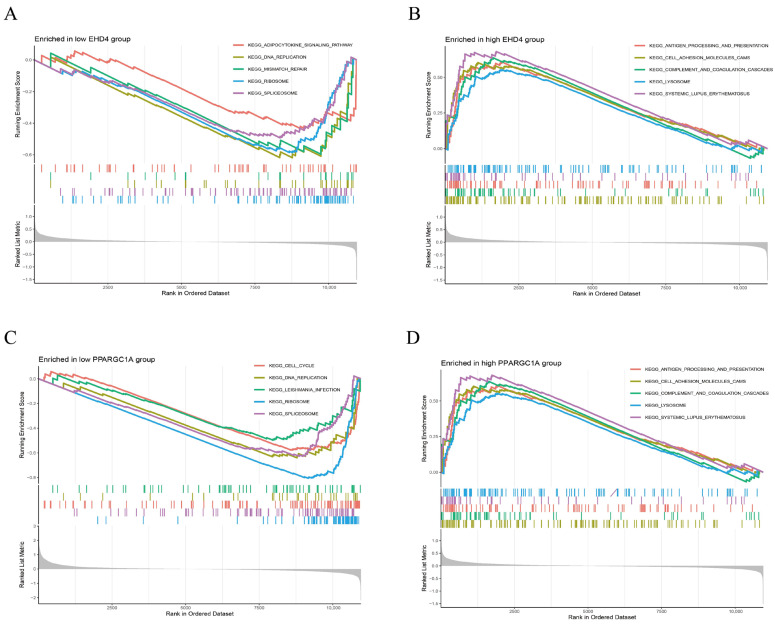
Gene set enrichment analysis (GSEA) reveals distinct KEGG pathway signatures in low- and high-expression groups of *EHD4* and *PPARGC1A*. (**A**): The top 5 active biological functions in the EHD4 low-expression group; (**B**): The top 5 active biological functions in the *EHD4* high-expression group; (**C**): The top 5 active biological functions in the *PPARGC1A* low-expression group; (**D**): The top 5 active biological functions in the *PPARGC1A* high-expression group.

We further performed GSEA analysis to reveal significant differences in the functional pathways associated with the differential expression levels of the key genes. In the low-*EHD4* expression group, the most enriched pathways included the adipocytokine signaling pathway, DNA replication, mismatch repair, ribosomes, and spliceosomes (Figure 6A). Conversely, in the high-*EHD4* expression group, the most enriched pathways were antigen processing and presentation, cell adhesion molecules, complement and coagulation cascades, lysosomes, and systemic lupus erythematosus (Figure 6B). For *PPARGC1A*, the low-expression group exhibited enriched pathways related to cell cycle, DNA replication, EBV infection, ribosomes, and spliceosomes (Figure 6C). In contrast, the high-*PPARGC1A* expression group was primarily enriched for pathways involving Complement and Coagulation Cascades, Drug Metabolism Cytochrome P450, Metabolism of Xenobiotics, Retinol Metabolism, and Valine, Leucine, and Isoleucine Degradation (Figure 6D).

### 3.5. Assessment of Immune Cell Infiltration in HCC

**Figure 7:**
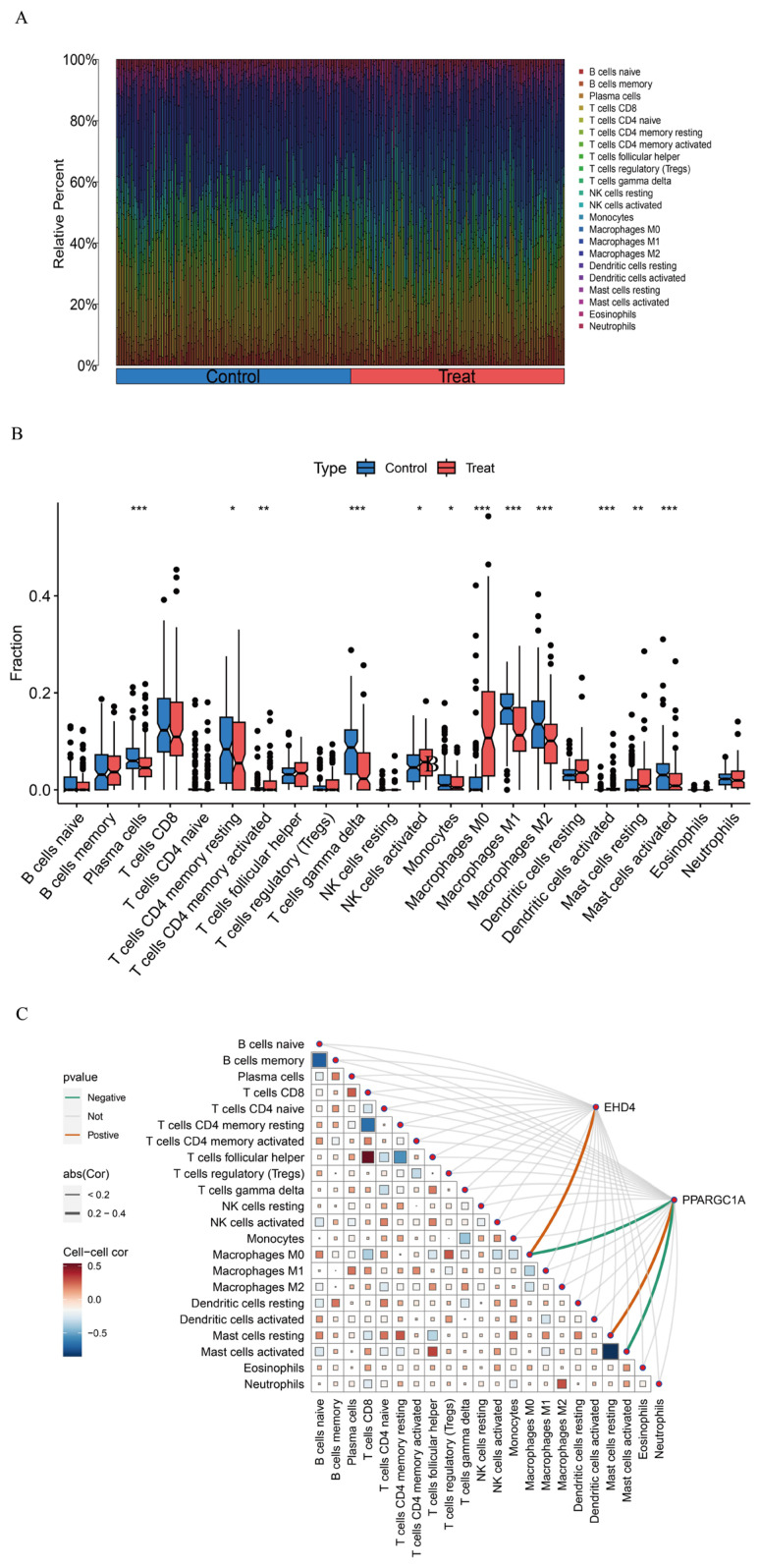
HCC immune landscape assessment. (**A**): Comparative immune cell composition displayed as vertical distribution bars comparing control versus HCC tissues; (**B**): Differential abundance of immune cell populations (n = 22) visualized through quartile distribution plots highlighting significant variations between normal and malignant liver samples; (**C**): Correlation matrix visualizing interactions between identified immune cell subtypes and key differentially expressed genes. Statistical significance threshold: * *p* < 0.05. In panel (**C**): The size of squares represents the absolute value of correlation coefficients, with larger squares indicating stronger correlations, and statistical significance is indicated by filled versus empty squares. Statistical significance threshold: * *p* < 0.05, ** *p* < 0.01, *** *p* < 0.001.

Gene function and pathway exploration suggested that the HCC co-expressed gene set is tightly linked to immune and inflammatory activities. Immune cell composition was therefore estimated with CIBERSORT, which deconvoluted the relative abundances of 22 leukocyte subsets for every sample (Figure 7A). Comparisons between tumor and non-tumor tissue uncovered marked shifts in several populations—most notably γδ T cells, activated dendritic cells, activated mast cells, plasma cells, and the M0, M1, and M2 macrophage subsets (Figure 7B)—implying that these cells may participate in HCC development. We next examined how the two hub genes relate to the immune landscape using the linkET package. *EHD4* showed a positive association with M0 macrophages, whereas *PPARGC1A* was inversely related to M0 macrophages and activated mast cells but positively linked to resting mast cells (Figure 7C). These patterns indicate that the two genes may influence macrophage polarization and mast cell activation in the tumor micro-environment.

### 3.6. Validation Group Differential Analysis of the Hub Genes

**Figure 8:**
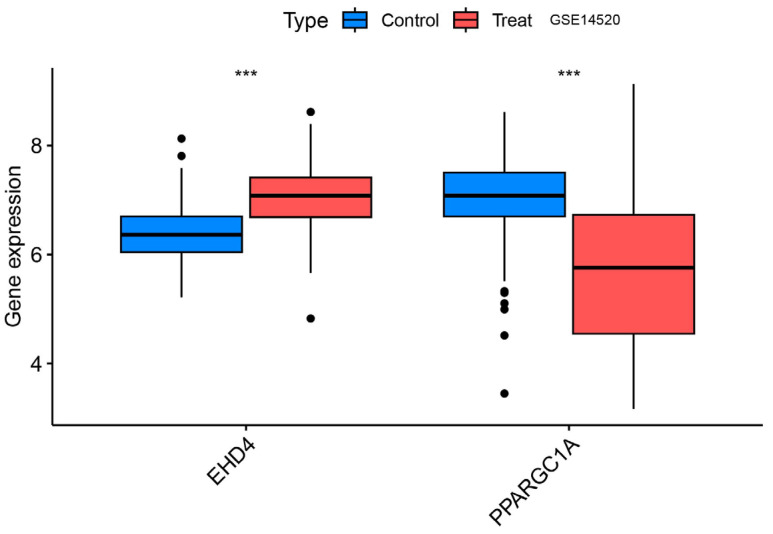
Validation group differential analysis of the hub genes. ***, *p* < 0.001.

We confirmed that the co-expressed genes identified in the MR analysis had the appropriate expression levels in the GSE14520 dataset. The results showed that *EHD4* expression was significantly higher in the HCC samples than in the healthy controls. *PPARGC1A* expression was significantly lower than in healthy controls (Figure 8), and the expression levels of both genes were consistent with our MR analysis results.

### 3.7. Expression Analysis of PPARGC1A and EHD4 in Rat HCC Model

**Figure 9:**
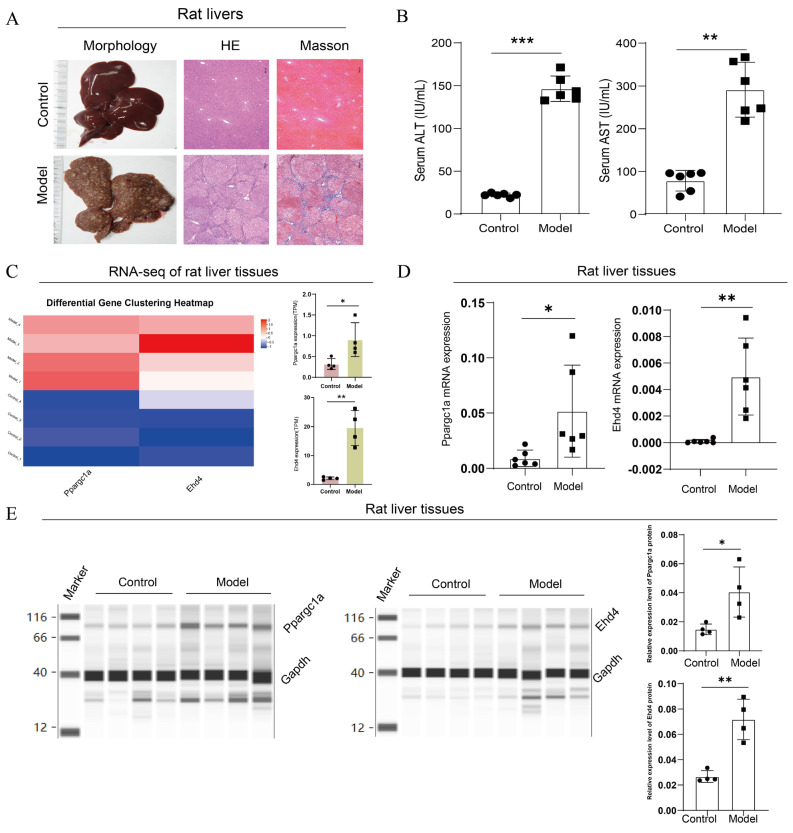
Upregulated expression of *PPARGC1A* and *EHD4* in rat HCC model. (**A**): Microscopic images of rat liver histopathology stained with H&E and Masson (50× magnification); (**B**): Serum levels of ALT and AST in rats; (**C**): Transcriptome analysis of rat livers. (**D**): Ppargc1a and Ehd4 mRNA expression in rat liver tissue; (**E**): Ppargc1a and Ehd4 protein expression in rat liver tissue. (**B**–**E**) Data were presented as the mean ± SD, and the *p* value was calculated by performing an unpaired Student’s *t*-test, *, *p* < 0.05; **, *p* < 0.01; ***, *p* < 0.001; ns, not significant.

Following the completion of the rat model, we first performed pathological and serum index detection. Compared with the control group, the H&E staining of the model group exhibited disrupted lobular structures with pseudo-lobules and hyperplastic nodules formed under fibrous encapsulation; the central veins were displaced, hepatocytes varied in size in the presence of multinucleated or binucleated cells, and the nuclear-to-cytoplasmic ratio increased; degeneration, necrosis, and dysplastic hyperplasia were observed in some of the hepatocytes; the arrangement of the hepatic cords was disordered; and there was marked fibrous proliferation in the portal areas, accompanied by inflammatory cell infiltration. Masson’s trichrome staining showed no significant blue staining in the control group, while in the model group, there was a substantial increase in collagen fiber proliferation, with fibers encapsulating pseudo-lobules and abnormal hyperplastic nodules (Figure 9A). The serum detection results showed that compared with the control, the ALT and AST levels were significantly upregulated in the model group (Figure 9B). These results suggested the successful construction of the rat HCC model.

Next, we analyzed the expression of the hub genes in rats’ livers. In the transcriptome analysis of rat livers, compared to the control group, we observed upregulation of *PPARGC1A* and *EHD4* genes in the model group (Figure 9C). To further validate this finding, we conducted qRT-PCR analysis of *Ppargc1a* and *Ehd4* genes in rat liver tissues, which revealed a significant increase in the model group compared to those in the control group (Figure 9D). Finally, we employed capillary protein electrophoresis analysis to assess protein expression in the rat liver, and the results indicated significant increases in Ppargc1a and *Ehd4* in the model group compared to the control (Figure 9E). These findings underscore the unique significance of these two targets in HCC.

### 3.8. Expression Analysis of EHD4 in Clinical Samples

**Figure 10:**
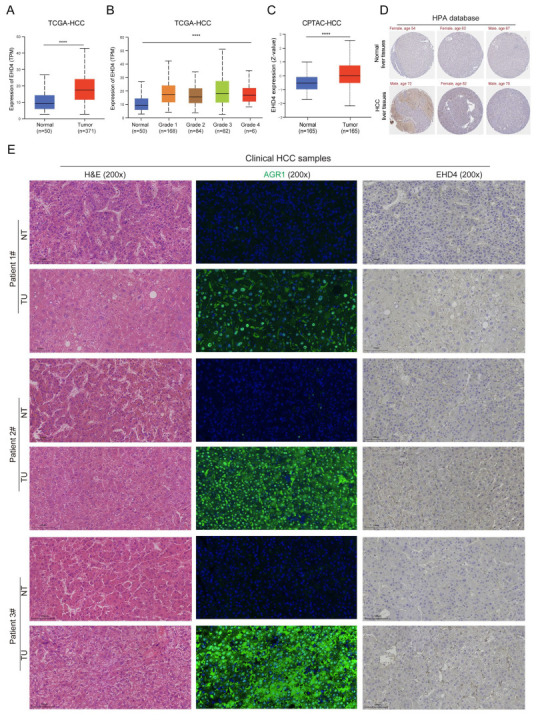
The expression of *EHD4* in HCC. (**A**) The mRNA expression of *EHD4* in the TCGA-HCC database according to sample type. (**B**) The mRNA expression of *EHD4* in the TCGA-HCC database according to tumor grade. (**C**) The protein expression of *EHD4* in the CPTAC database. (**D**) The IHC staining of *EHD4* in the HPA database. (**E**) The section staining of clinical HCC samples. ****, *p* < 0.0001.

The above results showed the importance of these two hub genes, and the expression trends of *EHD4* in public databases and the rat model were consistent. To further confirm the expression of *EHD4* in HCC, we analyzed the public databases and clinical samples. The results showed that in the TCGA database, *EHD4* mRNA expression is significantly upregulated in HCC compared to normal liver tissues (Figure 10A), and it is upregulated in tumor tissues with the increase in tumor grade (Figure 10B). In the CPTAC database, compared to the normal liver tissues, *EHD4* protein expression is significantly upregulated in HCC (Figure 10C), and the IHC staining also indicates the upregulation of *EHD4* in HCC in the HPA database (Figure 10D). Meanwhile, we collected three clinical HCC samples and detected the expression of *EHD4*. Our sample results showed that the protein expression of *EHD4* is upregulated in HCC tissues compared to non-tumor tissues (Figure 10E). Collectively, these findings indicate that the *EHD4* is a critical factor in the progression of HCC.

## 4. Discussion

In China, liver cancer ranked second among all cancers in terms of mortality in 2022, and the overall five-year survival rate remains low [15]. High mortality and low survival rates not only exacerbate the public health burden but also impose significant economic and social pressures [16]. Global projections indicate that if current prevention strategies remain unchanged, liver cancer deaths could increase by 55% by 2040 [3], further underscoring the urgency of early screening and comprehensive interventions. HCC evolves through chronic hepatitis, fibrosis, and cirrhosis in a step-wise, inflammation-driven cascade governed by multiple genetic alterations [17]. Persistent insults trigger chronic hepatic inflammation, which, after years of cumulative damage, can culminate in malignant transformation [18]. Mendelian randomization (MR) offers a unique approach to understanding the genetic underpinnings of liver cancer by leveraging genetic variants as instrumental variables to infer causality between risk factors and the disease. This study integrated GEO transcriptomic data with two-sample Mendelian randomization to first identify *EHD4* and *PPARGC1A* as key genes causally associated with HCC, which are significantly associated with liver cancer progression, indicating that genetic susceptibility plays an important role in the pathogenesis of liver cancer. Differential expression analysis and CIBERSORT analysis revealed that the expression levels of these two genes were significantly correlated with tumor-associated macrophage infiltration. The findings were subsequently validated in a DEN-induced rat HCC model.

*EHD4*, an important member of the EHD family, is the first extracellular matrix protein to contain an EH structural domain [19]. And *EHD4*, in turn, as a membrane transport regulatory protein, belongs to the EHD family of proteins along with *EHD1*, *EHD2,* and *EHD3*, all of which contain an EH domain at the C-terminus [20,21]. Related studies [22,23,24] have found that the EHD family is also involved in regulating cellular endocytosis, which is closely related to cancer. Endocytosis plays a key role in cell signalling, polarity, and adhesion, which are critical for cancer cell growth and metastasis. Studies have shown that members of the EHD family, such as *EHD2*, play an important role in inhibiting the migration and invasion of HCC cells [25]. Decreased *EHD2* expression correlates with higher tumor grade and metastasis rates, suggesting that *EHD4* may play a role in HCC through a similar mechanism. In addition, *EHD4* may be closely related to signalling pathways in HCC. For example, *EHD3* promotes cancer progression by affecting the Wnt/β-catenin signalling pathway [26]. This signalling pathway is also important in HCC as it is closely related to cell proliferation, apoptosis, and differentiation. Therefore, *EHD4* may influence the occurrence and development of HCC by regulating similar signalling pathways. Another noteworthy aspect is that *EHD4* may influence chemotherapy resistance in HCC cells; other members of the EHD family, such as *EHD1*, have been shown to influence cellular sensitivity to chemotherapeutic drugs in certain cancers by modulating intracellular drug accumulation and efflux [27]. Investigating whether *EHD4* has a similar function in HCC will help develop new anti-cancer treatment strategies, and the expression of EHD*4* protein in clear cell carcinoma is significantly higher than that in eosinophilic cell tumors, which is more conducive to future clinically effective targeted therapy for patients with related cancers [28]. Although there are limited studies on the specific role of *EHD4* in HCC, by drawing on the studies of other members of the EHD family, we can speculate that *EHD4* may affect the progression, metastasis, and chemoresistance of HCC through multiple mechanisms. This provides a strong direction for future in-depth studies on the role of *EHD4* in HCC, and may provide new targets for HCC treatment.

*PPARGC1A*, also known as PPAR coactivator 1-alpha (*PGC-1α*), plays a pivotal role in cellular metabolism and energy homeostasis. The protein encoded by this gene initiates multiple functions, including alpha-tubulin, estrogen receptor, and peroxisome proliferator-activated receptor-binding activities. It is involved in processes such as cellular response to hexose stimuli, cellular response to organic cyclic compounds, and regulation of smooth muscle cell proliferation [29,30]. Tumor-associated macrophages (TAMs) and tumor microenvironments (TME s) are key drivers of hepatocarcinogenesis and progression, and *PPARGC1A* has been identified as a TAM-associated prognostic genes in liver cancer [31]. *PPARGC1A* has also been shown to be closely associated with liver cancer [32] and other related metabolic diseases [33]. Immunotherapy and competitive endogenous RNA (ceRNA) are the main research hotspots in hepatocellular carcinoma. Zuo et al. [34] identified the target gene *PPARGC1A* in the prognosis-associated sub-network of ceRNAs and further established a risk prediction model, elucidating its key role in predicting the poor prognosis of liver cancer. They found a strong correlation between different types of tumor-infiltrating immune cells and immune checkpoints, providing new ideas for the study of the pathogenesis of liver cancer. In another case–control study [35], the investigators assessed the specific association between liver cancer risk and *PPARGC1A* polymorphisms in a Moroccan population, confirming that *PPARGC1A* serves as a common prognostic marker for hepatocellular carcinoma, and that the *PPARGC1A* rs8192678 polymorphism is strongly associated with an increased risk of hepatocellular carcinoma in the Moroccan population. Qian et al. [36] conducted an in-depth analysis of *PPARGC1A* using the Cancer Genome Atlas dataset to effectively assess the impact of *PPARGC1A* on the overall survival of liver cancer patients and discovered that *PPARGC1A* is a protective factor for liver cancer patients and ultimately better prevents and predicts liver cancer metastases through the development of a more effective high-throughput technology to identify metastasis inhibitors, which is supported by our Mendelian randomization study. Notably, *PPARGC1A* was up-regulated in our DEN-induced rat HCC model, whereas several large-scale human studies have documented a marked down-regulation of *PPARGC1A* in hepatocellular carcinoma and linked its low expression to poor prognosis [32]. In contrast, *PPARGC1A* is over-expressed in non-small-cell lung cancer, where it actively promotes metastatic dissemination [37]. These discordant findings emphasize the existence of tissue- and species-specific regulatory circuits and may also reflect differences in tumor stage. Moreover, tumor-micro-environment heterogeneity and metabolic stressors such as hypoxia can switch *PPARGC1A* between tumor-suppressive and tumor-promoting roles. Deblois and colleagues showed that the PGC-1/ERR axis exerts bidirectional effects depending on cellular context [38]. Together, these observations underscore the importance of integrating multi-species datasets and carefully considering biological context when translating results from animal models to the clinic. Future work should delineate the temporal dynamics of *PPARGC1A* expression across HCC progression and unravel how micro-environmental cues modulate its function.

Our study indicates that both *EHD4* and *PPARGC1A* play a co-regulatory role in macrophage M0 polarization. The involvement of *EHD4* and *PPARGC1A* in hepatocellular carcinoma may be mediated by the regulation of tumor-associated macrophages (TAMs) and other immune cells. The observed positive correlation between *EHD4* and M0-type macrophages suggests a specific function of *EHD4* in unpolarized macrophages, which exist in an inactivated state and have the potential to differentiate into either pro-inflammatory M1-type or pro-tumorigenic M2-type macrophages, depending on microenvironmental cues. Macrophages are critical components of the immune system and are responsible for pathogen phagocytosis and antigen presentation. Their endocytic capacity is essential for normal immune function [39]. *EHD4* may influence the behavior of M0-type macrophages, potentially promoting tumor progression and immune escape by modulating the macrophage endocytosis pathway or the membrane protein recycling process [40]. *PPARGC1A* is closely linked to the immunoregulatory functions of TAMs, particularly the suppression of proinflammatory signals within the tumor microenvironment. *PPARGC1A* affects the invasive and metastatic potential of HCC cells by influencing macrophage activity, suggesting that it may be a crucial molecular target for modulating the HCC microenvironment [34,41]. Studies have shown that *PPARGC1A* expression is negatively correlated with M0 macrophages, an undifferentiated form of macrophages in the TME. This correlation suggests that *PPARGC1A* may influence macrophage polarization, potentially reducing the pro-tumoral M2 phenotype, which is often associated with tumor progression in HCC [42]. Additionally, in hepatocellular carcinoma, *PPARGC1A* was positively correlated with resting mast cells and negatively correlated with activated mast cells, suggesting that *PPARGC1A* may play a key role in regulating the mast cell activation status in the tumor microenvironment. Mast cells play a complex role in tumor development and are usually involved in pro-inflammatory and immune regulation. The positive correlation between *PPARGC1A* and resting mast cells suggests that it may reduce the tumor-promoting effects of mast cells by maintaining the inactivated state of these cells. Meanwhile, the negative correlation between *PPARGC1A* and activated mast cells suggests that it may attenuate the pro-inflammatory effects of mast cells in the tumor microenvironment by inhibiting their activation [43,44,45]. *PPARGC1A* may play a protective role in limiting tumor progression by regulating mast cell status. Further studies on this mechanism may provide new targets for the treatment of hepatocellular carcinoma.

In summary, genome-wide association studies (GWASs) in recent years still provide relatively limited genetic evidence for *EHD4* and *PPARGC1A* in hepatocellular carcinoma. In the case of *PPARGC1A*, its classical coding variant rs8192678 (Gly482Ser) was reported to be associated with an increased risk of HCC in a case–control study in North African Morocco [35]. However, in a large North American multicenter GWAS from 2024, the *PPARGC1A* locus did not reach the genomics significance threshold, suggesting that the risk effect may be population- or environment-specific. As for *EHD4*, no significant association signals located at the *EHD4* locus have been detected in any of the currently published GWAS, including multi-stage cohorts of HBV-associated and non-viral HCC, which supports the importance of confirming *EHD4* function in the present study through causal extrapolation and animal studies. Eventually, our study identified *EHD4* and *PPARGC1A* as key regulators in hepatocellular carcinoma (HCC), particularly through their roles in modulating tumor-associated macrophages (TAMs) and other immune cells within the tumor microenvironment. The positive correlation between *EHD4* and M0-type macrophages underscores its potential function in influencing macrophage polarization and promoting tumor progression and immune evasion. Similarly, *PPARGC1A* appears to play a dual role in HCC, affecting both macrophage activity and the immune landscape. These findings not only deepen our understanding of the molecular mechanisms driving HCC but also highlight *EHD4* and *PPARGC1A* as promising targets for therapeutic intervention. Future studies should focus on validating these targets in a clinical setting and exploring their potential in developing personalized treatment strategies for HCC.

### Study Limitations

Although this study combined multi-omics data, Mendelian randomization, and animal model systems to reveal the potential role of *EHD4* and *PPARGC1A* in hepatocellular carcinoma, certain limitations remain. In vivo validation used a DEN-induced rat model, which differs from human HCC in genetic background and immune microenvironment, so extrapolation to clinical settings requires caution. The study lacks functional experimental evidence, such as gene knockout/overexpression experiments, making it difficult to fully elucidate the molecular mechanisms by which these genes regulate immune cells. Additionally, the cross-sectional design failed to dynamically track the expression changes in target genes across different stages of HCC. Future studies should include prospective follow-up in multi-ethnic population cohorts and combine gene editing technologies to conduct in-depth functional validation, thereby providing more reliable molecular targets for the clinical treatment of liver cancer.

## Figures and Tables

**Table 1:** The primers used in this study.

Primer Name	Primer Sequence (5′-3′)
**Gapdh**	F: ATGGGACGATGCTGGTACTGA R: TGCTGACAACCTTGAGTGAAAT
**Ehd4**	F: CCTGCGCTCTCTGTACCAG R: TCCCCATACATCACAGCAATGA
**Ppargc1a**	F: TATGGAGTGACATAGAGTGTGCT R: CCACTTCAATCCACCCAGAAAG

## Data Availability

The datasets generated and/or analyzed during the current study are available from the corresponding author upon reasonable request (yeyongan@vip.163.com).

## Supplementary Materials

The following supporting information can be downloaded at: https://www.mdpi.com/article/10.3390/biomedicines13061313/s1, Table S1: MR Analysis Results; Table S2: Heterogeneity Test Results for MR Analysis; Table S3: MR-Egger Intercept Test for Horizontal Pleiotropy.
